# Hospital Notes

**Published:** 1895-07

**Authors:** 


### Hospital Notes.

The Buffalo Fresh Air Mission Hospital will be opened July 1st, at Athol Springs, for the reception and treatment of young children suffering from cholera-infantum and other acute and dangerous diarrheal diseases. Last summer a new hospital, with thirty beds, was erected on a beautiful piece of land on the lake shore at Athol Springs. The plans are in accordance with the most approved system of summer hospital construction, and the institution is equipped with every modern appliance for the relief and care of all varieties of infantile diarrheal disorders.

A commodious administration house with diet kitchen and with rooms for the staff, nurses and help is attached to the hospital.

Dr. George J. Haller and Dr. W. J. Howells, house physicians, will reside at the hospital and the patients will be under continual medical surveillance. A corps of trained nurses, (hospital graduates,) under the superintendence of Miss Laura B. King, will have charge of the little patients. The staff, Drs. Snow and Sherman, will make daily visits to the hospital. All physicians are familiar with the beneficial effects of fresh country air and correct diet for acute infantile diarrheas. The physicians of Buffalo are urged to interest themselves in this worthy charity and to send to Athol Springs babies and young children attacked with cholera-infantum and other grave forms of diarrheal disease. Every baby must be accompanied by its mother, or by some adult person who will remain at the hospital during the child's illness.

For obvious reasons no contagious disease, such as whooping cough, scarlatina, measles or diphtheria, can be received. The hospital is on the lake shore, ten miles from the city, and is reached in a half hour by the Lake Shore or W. N. Y. & P. railroads. It is a five minutes' walk from the station at Athol Springs.

As it is especially desired that the little patients shall be sent at once into the country with no delay or vexatious formality for physicians, or parents, or friends, any one wishing to send a patient to the Fresh Air Mission Hospital should go, or telephone, to the Fitch Institute, or to the residence of Dr. Snow or Dr. Sherman, or the sick child may be brought to the University Dispensary, 24 High street, between 10 and 11 A. M. Information and tickets will be furnished at these places.

It should be expressly understood that no charge is made for either baby or mother. All trains for Athol Springs leave the New York Central station on Exchange street.

Physicians are cordially invited to inspect the hospital.

---

AT THE Buffalo General Hospital, Dr. Sidney D. Wilgus has been appointed junior house surgeon and Dr. W. Grant Cooper junior house physician. Drs. N. Gorham Russell and George J. Haller will succeed to the above places October 1, 1895, when Drs. Wilgus and Cooper will become senior house surgeon and house physician respectively.

At the Fitch Emergency Hospital, Drs. Carl C. Mann, B. Ross Nairn and A. E. Dean have been appointed house surgeons. All of the above-mentioned physicians are graduates of Buffalo University Medical College in class of '95.

---

AT THE Hospital of the Sisters of Charity, Drs. Arthur V. Conley, Bion E. Smith and William C. Lewin have been appointed internes after a competitive examination. Dr. Bernard H. Brady has been appointed house surgeon at the Emergency Hospital also as the result of a similar test. These young men are all graduates of Niagara University in the class of '95.

